# Salivary antibody responses to *Fusobacterium nucleatum* and *Candida albicans* as indicators of periodontitis severity

**DOI:** 10.34172/japid.026.3982

**Published:** 2026-04-22

**Authors:** Muhammad Ihsan Rizal, Endang Winiati Bachtiar, Boy Muchlis Bachtiar, Yuniarti Soeroso, Fatimah Maria Tadjoedin, Retno Damajanti Soejoedono, Ferry Pergamus Gultom, Natalina Haerani, Melanie Sadono Djamil, Boedi Oetomo Roeslan

**Affiliations:** ^1^Department of Oral Biology, Faculty of Dentistry, Universitas Trisakti, Jakarta, Indonesia; ^2^Center of Molecular Biology Study, Faculty of Dentistry, Universitas Trisakti, Jakarta, Indonesia; ^3^Department of Oral Biology, Faculty of Dentistry, Universitas Indonesia, Jakarta, Indonesia; ^4^Department of Periodontology, Faculty of Dentistry, Universitas Indonesia, Jakarta, Indonesia; ^5^Department of Animal Diseases and Veterinary Health, Medical Microbiology Division, Faculty of Veterinary Medicine, IPB University, Bogor, Indonesia

**Keywords:** Humoral immune response, Keystone pathogen, Periodontitis, Polymicrobial, Saliva

## Abstract

**Introduction:**

Periodontitis is a chronic inflammatory disease that leads to the destruction of tooth-supporting tissues and remains a major cause of tooth loss worldwide. Its pathogenesis involves complex interactions between microbial pathogens and host immune responses, where salivary immunoglobulins serve as a first line of defense at mucosal surfaces. Identifying pathogen-specific salivary antibody responses that correlate with disease severity may provide noninvasive biomarkers for diagnosis and monitoring. The present work sought to explore how selected microbial pathogens and salivary antibody responses are connected with the severity of periodontitis and clinical periodontal status.

**Methods:**

A cross-sectional approach was applied. Saliva was obtained from 39 participants, including 31 patients diagnosed with periodontitis and 8 individuals with healthy periodontal tissues. Western blotting and enzyme-linked immunoassays were used to detect both microorganisms and immunoglobulins.

**Results:**

Patients classified as Stage IV periodontitis showed the highest occurrence of *Aggregatibacter actinomycetemcomitans*. Elevated IgA antibodies against *Fusobacterium nucleatum* (*P*=0.014) and *Candida albicans* (*P*=0.009) demonstrated significant associations with disease severity. Further associations were observed: plaque index with IgG to *C. albicans*; oral hygiene index with IgA to *A. actinomycetemcomitans* (*P*=0.008) and *C. albicans* (*P*=0.031), and papillary bleeding index with IgA to *A. actinomycetemcomitans* (*P*=0.003), *F. nucleatum* (*P*=0.002), and *C. albicans* (*P*=0.008).

**Conclusion:**

Salivary IgA and IgG responses to *Fusobacterium nucleatum* and *Candida albicans* exhibited significant stage-related associations with periodontitis severity, supporting their potential role as complementary immunological indicators.

## Graphical Abstract

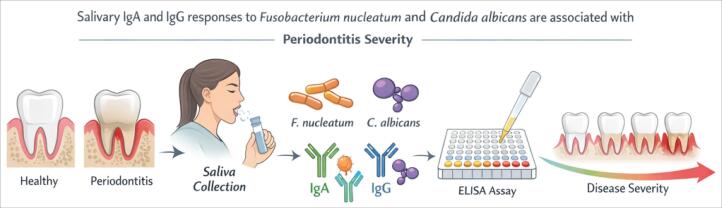

## Introduction

 The condition known as periodontitis involves progressive destruction of the structures that support teeth, such as the periodontal ligament, gingival tissues, cementum, and alveolar bone.^1,2^ Data from the World Health Organization highlight this disorder as a major contributor to tooth loss, affecting roughly one-fifth of the adult population globally.^3^ Other epidemiological evidence has suggested a wider prevalence, estimated between 20% and 50% worldwide.^4^ The Global Burden of Disease Study (2016) placed severe forms of periodontal disease as the sixth most widespread health problem globally.^5^ As scientific understanding of its pathogenesis has deepened, and definitions and classifications of periodontitis have been repeatedly updated. The most recent revision, issued in 2017, stratifies cases into four stages—ranging from Stage I through Stage IV—according to both clinical severity and treatment considerations.^6^

 The pathogenesis of periodontitis has been interpreted through multiple conceptual models, including nonspecific and specific plaque hypotheses, the ecological plaque perspective, the keystone pathogen model, and most recently, the polymicrobial synergy and dysbiosis (PSD) framework. The keystone pathogen concept emphasizes that certain microbes, even when present at low abundance, can reprogram the oral microbiota toward dysbiosis and undermine host defenses, in part by deriving sustenance from epithelial cells.^7^ Central to this idea is the ability of such organisms to interfere with immune function, especially with neutrophils positioned between dental biofilms and the epithelial layer. *Porphyromonas gingivalis* has long been regarded as the archetypal keystone pathogen in periodontitis.^8^ Later, Lamont et al.^9^ refined this concept into the PSD hypothesis.^9^ Findings from animal experiments confirmed that mixed infections by periopathogens exhibit markedly stronger pathogenicity compared with single-species infections. This increased virulence likely reflects the requirement of multiple bacterial factors—including adhesins, receptors, proteases, and proinflammatory surface molecules—that are absent in single keystone strains. Acting together, these elements constitute a collective virulence trait that promotes persistent dysbiosis, triggers inflammatory responses, and ultimately contributes to periodontal tissue breakdown.^10^

 In an investigation of salivary proteomics in periodontitis, six proteins were found to be elevated in individuals with aggressive disease compared with healthy controls: serum albumin, immunoglobulin A (IgA), immunoglobulin G (IgG), vitamin D-binding protein, salivary α-amylase, and zinc-α2 glycoprotein.^11^ IgA contributes significantly to defense against bacterial and viral pathogens, while IgG, a serum-derived antibody, specifically targets microorganisms implicated in periodontal disease. Both immunoglobulins arise from the adaptive immune response, which is orchestrated by activated B and T lymphocytes.^12^ These lymphocytes express receptors that recognize antigens—unique microbial or host-derived molecules—thereby enabling the immune system to respond with remarkable specificity.^13^

 Within salivary glands, plasma cells produce IgA, making it the most prevalent antibody in saliva.^14,15^ IgG, in contrast, is mainly synthesized within gingival tissues; during inflammation, B lymphocytes differentiate into plasma cells and release IgG locally. Circulating antibodies can also access the oral cavity through gingival crevicular fluid. In addition to immunoglobulins, saliva contains multiple innate antimicrobial factors secreted by salivary glands, epithelial cells, and neutrophils. These substances restrict or eliminate bacterial, fungal, and viral growth.^13^

 Despite growing interest in salivary biomarkers in periodontitis, most previous investigations have focused on single microorganisms or isolated host markers, often without integrating microbial detection with humoral immune responses across clinically stratified disease stages. For this purpose, the following microorganisms were chosen as representative antigens known to elicit adaptive immune reactions: *Aggregatibacter actinomycetemcomitans*, *Treponema denticola*, *Porphyromonas gingivalis*, *Fusobacterium nucleatum*, and *Candida albicans*. Furthermore, few studies have evaluated salivary antibody profiles in the context of the 2017 AAP/EFP classification system, which provides a more refined staging framework for disease severity. As a result, the relationship between polymicrobial presence, salivary immunoglobulin responses, and stage-specific clinical parameters remains insufficiently characterized. Therefore, this study aimed to evaluate salivary humoral immunologic markers and microbial presence across severity stages of periodontitis defined by the 2017 AAP/EFP classification.

## Methods

 This investigation followed an observational, cross-sectional design. It was carried out at three locations: the Dental Hospital of Universitas Indonesia, the Biomolecular Laboratory of the Faculty of Dentistry at Universitas Indonesia, and the BioCORE Laboratory of the Faculty of Dentistry at Universitas Trisakti. A priori sample size estimation was conducted using the Lemeshow formula for unpaired numerical analysis. With a significance level set at 5% (α = 0.05) and statistical power of 80%, and based on the anticipated standard deviation and expected mean difference between groups, the minimum sample size required was five subjects per group. Eligible participants were men and women aged 18–60 years, who visited the Universitas Indonesia Dental Hospital. The subjects were categorized as periodontitis patients (Stages I–IV) or individuals with clinically healthy periodontium. Only individuals willing to participate and provide written informed consent were enrolled. Participants were excluded if they had a history of systemic diseases, including hypertension, cardiovascular disease, kidney disease, or hematological disorders; had consumed systemic medications within the past three months; were pregnant or breastfeeding; or had received dental treatment within the previous three months.

 This study was approved by the Ethics Committee of the Faculty of Dentistry, Universitas Indonesia (Approval No. 17/Ethical Approval/FKGUI/III/2019; Protocol No. 070210219). All the participants provided written informed consent prior to enrolment. Demographic data were recorded, and each subject underwent a general health evaluation and a comprehensive periodontal examination. The following parameters were documented: plaque index, calculus index, papillary bleeding index, oral hygiene index, pocket depth, gingival recession, clinical attachment loss, and tooth count. Classification of disease severity followed the 2017 framework jointly proposed by the American Academy of Periodontology (AAP) and the European Federation of Periodontology (EFP).

###  Salivary Protein Preparation

 Unstimulated morning salivary samples (volume: < 2 mL) were collected into 15-mL tubes. To minimize protein degradation, samples were immediately placed on ice, and 50 µL of a protease inhibitor cocktail was added. Homogenization was performed by centrifugation (1300 × g, 5 min, 4°C). Supernatant aliquots (150 µL) were transferred into new microtubes and subjected to protein extraction using Genezol^TM^ reagent (GeneAid, Taiwan). Salivary protein samples were stored at -20°C prior to analysis to preserve protein integrity. Protein concentrations were then determined with the Bradford assay (Bio-Rad, USA), according to the manufacturer’s protocol.

###  Western Blotting

 Western blotting was performed under denaturing and reducing conditions using SDS-PAGE, followed by semi-dry transfer to PVDF membranes and indirect HRP-based chromogenic detection. Rabbit polyclonal antibodies against *Aggregatibacter actinomycetemcomitans*, *Treponema denticola*, *Porphyromonas gingivalis*, *Fusobacterium nucleatum*, and *Candida albicans* were generated at the Faculty of Veterinary Medicine, IPB University. These antibodies had been purified previously using a high-affinity purification kit (GenScript, USA) and validated for specificity through dot blotting. Protein samples ( ≤ 3 μg) were mixed with buffer (Bio-Rad, USA) and β-mercaptoethanol (Sigma-Aldrich, USA) at a 19:1 ratio and then diluted 1:1 with sample buffer to a final volume of 20 μL. Following denaturation at 100°C for 5 min, 20 μL of each preparation was loaded into polyacrylamide gels along with pre-stained molecular weight standards (Precision Plus Protein^TM^ Dual Color Standards, Bio-Rad, USA). Electrophoresis was conducted at 90 V for 15 min and then at 125 V for 1 h. Proteins were transferred to PVDF membranes using a Trans-Blot Turbo semi-dry system (Bio-Rad, USA). Goat anti-rabbit IgG conjugated to HRP (1:1000, 5% non-fat milk; Thermo Fisher Scientific, USA) served as the secondary antibody. Protein visualization was achieved using the 1-Step^TM^ Chloronaphthol Substrate Solution (Thermo Fisher Scientific, USA), and band intensities were quantified with ImageJ software.

###  ELISA for Salivary IgA and IgG

 Indirect enzyme-linked immunoassays (ELISA) were performed to assess IgA and IgG reactivity against the selected pathogens. Wells were first coated with 100 μL of carbonate buffer (3.7 g of sodium bicarbonate and 0.64 g of sodium carbonate in 1 L of distilled water). Pathogens were added in 50-μL aliquots at the following concentrations: *A. actinomycetemcomitans*, 5.4 × 10⁷ CFU/mL; *T. denticola* (ATCC® 35405), 2 × 10⁸ CFU/mL; *P. gingivalis* (ATCC® 33277), 1.3 × 10⁷ CFU/mL; *F. nucleatum* (ATCC® 25586), 3.89 × 10⁸ CFU/mL; and *C. albicans* (ATCC® 10231), 5.9 × 10⁷ CFU/mL. Plates were incubated overnight at 4°C with 5% CO₂. After incubation, the wells were emptied and washed three times with 200 μL of TBS-T buffer. Blocking was performed with 150 μL of 5% non-fat milk for 1 h at 37°C and 5% CO₂. After washing, 50 μL of saliva from each participant was added and incubated for 1 h under the same conditions. The plates were washed again, and 100 μL of HRP-conjugated secondary antibodies (anti-human IgA and anti-human IgG, 1:2000 in 5% non-fat milk; Thermo Fisher Scientific, USA) was added. Absorbance was measured at 450 nm using an iMark^TM^ Microplate Reader (Bio-Rad, USA). Cut-off values for IgA and IgG responses were determined using receiver operating characteristic (ROC) curve analysis.

###  Statistical Analysis

 Data were analyzed using SPSS® 23. Descriptive statistics summarized demographic and clinical characteristics. Normality of continuous variables, including clinical indices, salivary protein concentration, and antibody optical density values, was assessed using the Shapiro–Wilk test. Inter-group comparisons across periodontitis stages were performed using one-way ANOVA with post hoc Bonferroni correction for normally distributed data and the Kruskal–Wallis test followed by the Mann–Whitney U test for non-normally distributed data. Associations between categorical variables, including antibody response categories and clinical index classifications, were evaluated using the chi-squared test, with odds ratios (OR) and 95% confidence intervals (CI) calculated where appropriate. Spearman’s rank correlation was specifically applied to assess the relationship between the presence of *Aggregatibacter actinomycetemcomitans* antigen and salivary IgA and IgG responses in Stage III periodontitis subjects. Statistical significance was set at P < 0.05.

## Results

 Thirty-nine individuals were included in this study, 12 of whom were men (30.7%) and 27 were women (69.2%). Using the 2017 AAP/EFP classification, 31 participants were diagnosed with periodontitis at different stages, while eight were periodontally healthy. As detailed in Table 1, subjects with more advanced stages tended to be older, with mean age rising from 25.1 ± 2.9 years in the healthy group to 51.0 ± 6.1 years in Stage IV. Indices reflecting oral health status showed a clear gradient: plaque index, oral hygiene index, and papillary bleeding index increased progressively with disease severity. For instance, the plaque index rose from 0.45 ± 0.13 in healthy subjects to 1.58 ± 0.30 in Stage IV. By contrast, salivary protein concentrations remained relatively stable, ranging from 147.38 ± 4.21 to 158.38 ± 12.10 µg/mL, suggesting that total protein content in saliva was not influenced by disease stage.

**Table 1:** Characteristics of participants stratified by periodontitis severity. Variables include gender distribution, mean age, plaque index, oral hygiene index, papillary bleeding index, and salivary protein concentration

**Periodontitis Stage (n)**	**Gender ** **(M/F)**	**Mean Age** **±SD**	**Mean PI** **±SD**	**Mean OHI ** **±SD**	**Mean PBI ** **±SD**	**Salivary protein ** **±SD (µg/mL)**
Healthy (8)	1/7	25.1 ± 2.9	0.45 ± 0.13	0.25 ± 0.21	0.10 ± 0.09	147.38 ± 4.21
Stage I (5)	2/3	41.8 ± 5.3	0.69 ± 0.25	1.05 ± 0.47	0.67 ± 0.45	153.24 ± 4.55
Stage II (8)	3/5	45.1 ± 7.3	0.82 ± 0.27	1.33 ± 0.45	0.92 ± 0.59	156.37 ± 11.34
Stage III (11)	4/7	45.9 ± 8.3	1.27 ± 0.35	2.68 ± 0.57	1.67 ± 0.54	158.38 ± 12.10
Stage IV (7)	2/5	51.0 ± 6.1	1.58 ± 0.30	3.48 ± 0.54	2.59 ± 0.25	155.57 ± 7.03

SD: standard deviation; M: male; F: female; PI: plaque index; OHI: oral hygiene index; PBI: papilla bleeding index

###  Detection of A. actinomycetemcomitans

**Figure 1:**
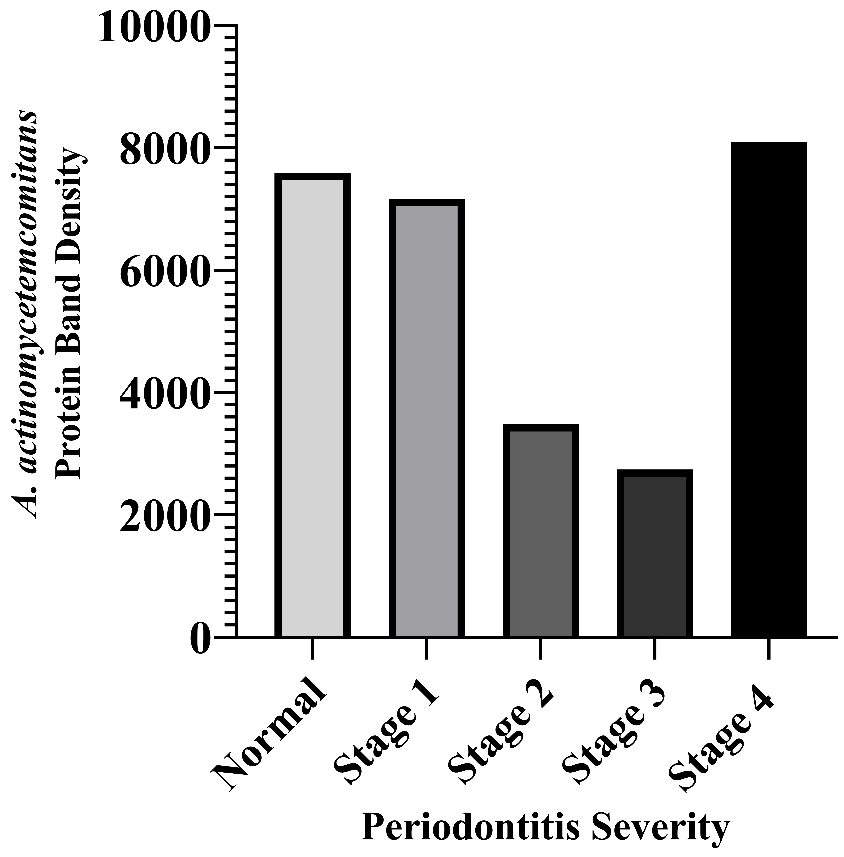
ImageJ analysis of protein band density in the western blot assay of *Aggregatibacter actinomycetemcomitans* antigen in saliva based on periodontitis severity. Median (min-max) normal: 7594.34 (1867.27-11343.25); Stage I: 7170.35 (1417.93-14,639.41); Stage II: 3482.51 (600.33-20.242.59); Stage III: 2742.23 (1009.98-10925.25); Stage IV: 8096.35 (1134.08-14,587.27). Kruskal-Wallis test *P* = 0.453

 Western blot analysis identified *A. actinomycetemcomitans* antigen in the saliva of all study participants. Quantification of band density using ImageJ software revealed the highest median values among Stage IV cases (8096.35; range: 1134.08–14587.27), compared with 7594.34 in the healthy group. However, this apparent trend was not statistically significant (Kruskal–Wallis, *P* = 0.453) (Figure 1), indicating that although *A. actinomycetemcomitans* was present across groups, its abundance alone may not directly reflect disease severity.

###  Salivary IgA Responses

**Figure 2:**
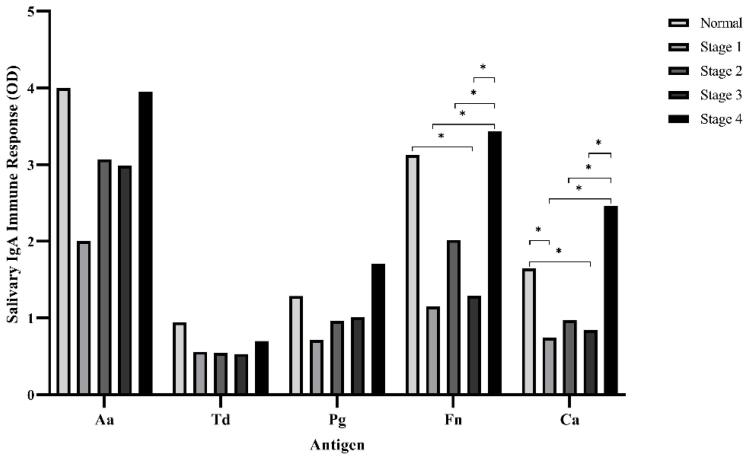
Median values of salivary IgA immune response to periodontal bacterial antigens and *Candida albicans* based on periodontitis severity. Kruskal‒Wallis test and post hoc Mann‒Whitney U test (* = significantly different; *P* < 0.05). Aa: *Aggregatibacter actinomycetemcomitans*; Td: *Treponema denticola*; Pg: *Porphyromonas gingivalis*; Fn: *Fusobacterium nucleatum*; Ca: *Candida albicans*; OD: optical density

 The analysis of IgA responses demonstrated stage-dependent variability. Significant associations were observed for *F. nucleatum* (*P* = 0.014) and *C. albicans* (*P* = 0.009), with antibody levels peaking in Stage IV subjects (Figure 2). Median IgA optical density values for *F. nucleatum* ranged from 1.15 in Stage I to 3.44 in Stage IV, while those for *C. albicans* increased from 0.74 in Stage I to 2.46 in Stage IV. In contrast, IgA responses to *A. actinomycetemcomitans*, *T. denticola*, and *P. gingivalis* did not differ significantly between the groups, suggesting that the IgA response may be more selectively amplified against certain pathogens.

###  Salivary IgG Responses

**Figure 3:**
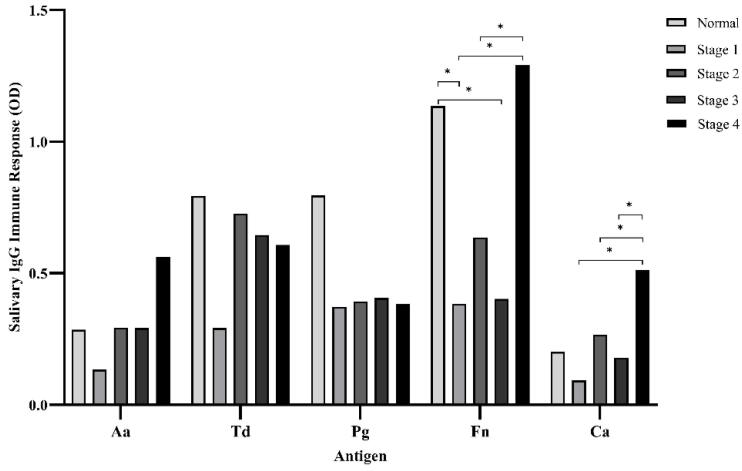
Median values of salivary IgG immune response to periodontal bacterial antigens and *Candida albicans* based on periodontitis severity. Kruskal–Wallis test and post hoc Mann-Whitney U test (* = significantly different; *P* < 0.05). Aa: *Aggregatibacter actinomycetemcomitans*; Td: *Treponema denticola*; Pg: *Porphyromonas gingivalis*; Fn: *Fusobacterium nucleatum*; Ca: *Candida albicans*; OD: optical density

 A similar pattern was found for IgG. Responses to *F. nucleatum* (*P* = 0.011) and *C. albicans* (*P* = 0.015) increased significantly with disease severity (Figure 3). In Stage IV patients, IgG reactivity reached median optical density values of 1.29 for *F. nucleatum* and 0.51 for *C. albicans*, compared with 1.14 and 0.20, respectively, in healthy controls. No significant stage-related differences were observed in IgG responses to *A. actinomycetemcomitans*, *T. denticola*, or *P. gingivalis*. These findings suggest that both IgA and IgG immune responses to *F. nucleatum* and *C. albicans* are closely linked to disease progression.

###  Correlation of A. actinomycetemcomitans with Antibody Responses

**Figure 4:**
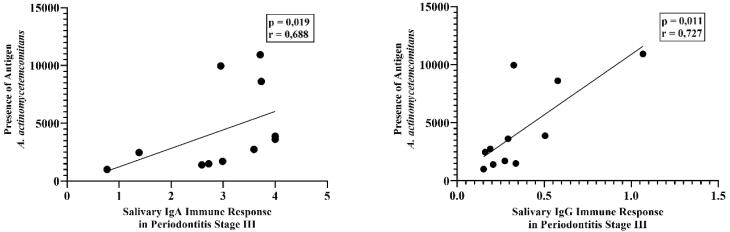
Relationship between the presence of *Aggregatibacter actinomycetemcomitans* antigen with IgA (left) and IgG (right) salivary immune responses in periodontitis stage III subjects. Description: Spearman’s test; IgA: immunoglobulin A; IgG: immunoglobulin G

 To explore whether bacterial presence was related to salivary antibody responses, Spearman’s test was performed. In Stage III subjects, the presence of *A. actinomycetemcomitans* showed strong positive correlations with both IgA and IgG responses (Figure 4). This finding suggests that the bacterium may stimulate local antibody production, particularly in intermediate stages of disease, when teeth are still present and immune interaction is active.

###  Clinical Indices and Disease Severity

**Figure 5:**
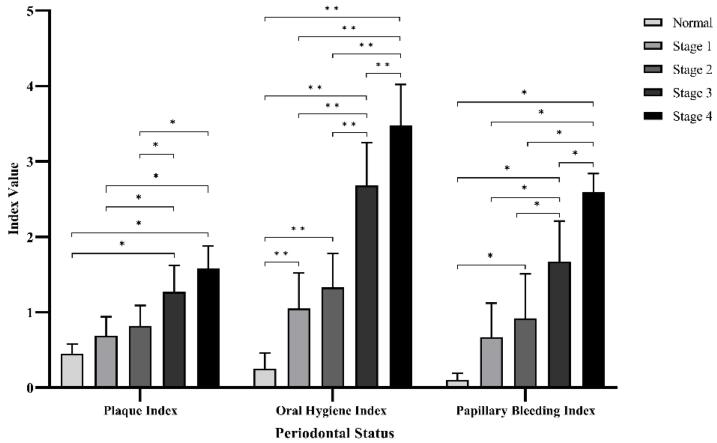
Mean values of periodontal status (plaque index, oral hygiene index, and papillary bleeding index) in periodontitis severity. ANOVA test and post hoc Bonferroni test (* = significantly different; *P* < 0.05). Kruskal–Wallis Test and post hoc Mann‒Whitney U Test (** = significantly different; *P* < 0.05)

 Comparison of clinical indices confirmed significant differences across disease stages. Plaque index, oral hygiene index, and papillary bleeding index were all strongly associated with severity (ANOVA/Kruskal–Wallis, all *P* < 0.001) (Figure 5). These indices therefore reflect not only disease stage but also the host’s inflammatory status.

###  Clinical Indices and Immune Responses

 Further analysis was performed to assess whether clinical indices were linked to salivary immune responses. As presented in Table 2, plaque index was significantly correlated with IgG reactivity to *C. albicans* (*P* = 0.041, OR = 5.333, 95% CI: 1.175–24.213), suggesting that increased plaque accumulation may enhance the humoral response to this fungal pathogen. In contrast, no statistically significant differences were found for other pathogens in the IgG analysis, nor were any significant associations observed in the IgA responses (*P* > 0.05).

**Table 2:** Correlation between plaque category and salivary IgA/IgG responses to selected periodontal pathogens

**Antibody**	**Pathogens**	**High response** **n (%)**	**Low response** **n (%)**	**Plaque** **category**	***P*** **-value**	**OR**	**95% CI**
IgA	*A. actinomycetemcomitans*	6 (54.5)/7 (25.0)	5 (45.5)/21 (75.0)	Thin vs. Moderate	0.131	0.278	0.064–1.200
	*T. denticola*	9 (45.0)/4 (21.1)	11 (55.0)/15 (78.9)	Thin vs. Moderate	0.176	0.326	0.079–1.337
	*P. gingivalis*	2 (20.0)/11 (37.9)	8 (80.0)/18 (62.1)	Thin vs. Moderate	0.445	2.444	0.437–13.672
	*F. nucleatum*	9 (45.0)/4 (21.1)	11 (55.0)/15 (78.9)	Thin vs. Moderate	0.176	0.326	0.079–1.337
	*C. albicans*	8 (44.4)/5 (23.8)	10 (55.6)/16 (76.2)	Thin vs. Moderate	0.196	0.391	0.099–1.535
IgG	*A. actinomycetemcomitans*	4 (19.0)/9 (50.0)	17 (81.0)/9 (50.0)	Thin vs. Moderate	0.087	4.250	1.019–17.729
	*T. denticola*	7 (26.9)/6 (46.2)	19 (73.1)/7 (53.8)	Thin vs. Moderate	0.290	2.327	0.578–9.367
	*P. gingivalis*	8 (50.0)/5 (21.7)	8 (50.0)/18 (78.3)	Thin vs. Moderate	0.090	0.278	0.069–1.119
	*F. nucleatum*	9 (42.9)/4 (22.2)	12 (57.1)/14 (77.8)	Thin vs. Moderate	0.307	0.381	0.093–1.557
	*C. albicans*	3 (15.8)/10 (50.0)	16 (84.2)/10 (50.0)	Thin vs. Moderate	**0.041**	5.333	1.175–24.213

OR: odds ratio; CI: confidence interval; chi-squared test significant values (*P* < 0.05) are shown in bold

 Oral hygiene index demonstrated significant associations with IgA responses to *A. actinomycetemcomitans* (*P* = 0.008, OR = 0.112, 95% CI: 0.022–0.567) and *C. albicans* (*P* = 0.031, OR = 0.153, 95% CI: 0.027–0.854) (Table 3). Poorer oral hygiene was thus linked to stronger local antibody responses against these organisms.

**Table 3:** Correlation between oral hygiene category and salivary IgA/IgG responses to selected periodontal pathogens

**Antibody**	**Pathogens**	**High response** **n (%)**	**Low response** **n (%)**	**Oral hygiene** **category**	**P-value**	**OR**	**95% CI**
IgA	*A. actinomycetemcomitans*	7 (53.8)/3 (11.5)	6 (46.2)/23 (88.5)	Good vs. Fair–Poor	**0.008**	0.112	0.022–0.567
	*T. denticola*	8 (40.0)/2 (10.5)	12 (60.0)/17 (89.5)	Good vs. Fair–Poor	0.065	0.176	0.032–0.982
	*P. gingivalis*	9 (37.5)/1 (6.7)	15 (62.5)/14 (93.3)	Good vs. Fair–Poor	0.057	0.119	0.013–1.064
	*F. nucleatum*	8 (40.0)/2 (10.5)	12 (60.0)/17 (89.5)	Good vs. Fair–Poor	0.065	0.176	0.032–0.982
	*C. albicans*	8 (42.1)/2 (10.0)	11 (57.9)/18 (90.0)	Good vs. Fair–Poor	**0.031**	0.153	0.027–0.854
IgG	*A. actinomycetemcomitans*	3 (17.6)/7 (31.8)	14 (82.4)/15 (68.2)	Good vs. Fair–Poor	0.464	2.187	0.469–10.119
	*T. denticola*	7 (38.9)/3 (14.3)	11 (61.1)/18 (85.7)	Good vs. Fair–Poor	0.141	0.262	0.056–1.230
	*P. gingivalis*	6 (37.5)/4 (17.4)	10 (62.5)/19 (82.6)	Good vs. Fair–Poor	0.264	0.351	0.080–1.540
	*F. nucleatum*	8 (40.0)/2 (10.5)	12 (60.0)/17 (89.5)	Good vs. Fair–Poor	0.065	0.176	0.032–0.982
	*C. albicans*	3 (16.7)/7 (33.3)	15 (83.3)/14 (66.7)	Good vs. Fair–Poor	0.290	2.500	0.538–11.617

*OR: odds ratio; CI: confidence interval; chi-squared test significant values (*P* < 0.05) are shown in bold

 Papillary bleeding index showed even broader associations, being significantly related to IgA responses against *A. actinomycetemcomitans* (*P* = 0.003, OR = 0.071), *F. nucleatum* (*P* = 0.020, OR = 0.083), and *C. albicans* (*P* = 0.008, OR = 0.072) (Table 4). In contrast, IgG responses did not show significant correlations with papillary bleeding. This indicates that IgA, as the predominant mucosal antibody, may play a more direct role in the local inflammatory processes associated with gingival bleeding. For all analyses, odds ratios below 1.0 reflect inverse associations based on the selected reference categories and should not be interpreted as protective effects.

**Table 4:** Correlation between papillary bleeding category and salivary IgA/IgG responses to selected periodontal pathogens

**Antibody**	**Pathogens**	**High response** **n (%)**	**Low response** **n (%)**	**Papillary bleeding** **category**	**P-value**	**OR**	**95% CI**
IgA	*A. actinomycetemcomitans*	7 (53.8)/2 (7.7)	6 (46.2)/24 (92.3)	Good vs. Fair–Poor	**0.003**	0.071	0.012–0.436
	*T. denticola*	7 (38.9)/2 (9.5)	11 (61.1)/19 (90.5)	Good vs. Fair–Poor	0.055	0.165	0.029–0.941
	*P. gingivalis*	8 (34.8)/1 (6.3)	15 (65.2)/15 (93.8)	Good vs. Fair–Poor	0.056	0.125	0.014–1.127
	*F. nucleatum*	8 (40.0)/1 (5.3)	12 (60.0)/18 (94.7)	Good vs. Fair–Poor	**0.020**	0.083	0.009–0.775
	*C. albicans*	8 (42.1)/1 (5.0)	11 (57.9)/19 (95.0)	Good vs. Fair–Poor	**0.008**	0.072	0.008–0.658
IgG	*A. actinomycetemcomitans*	6 (26.1)/2 (13.3)	17 (73.9)/13 (86.7)	Good vs. Fair–Poor	0.440	0.436	0.075–2.523
	*T. denticola*	7 (38.9)/2 (9.5)	11 (61.1)/19 (90.5)	Good vs. Fair–Poor	0.055	0.165	0.029–0.941
	*P. gingivalis*	4 (40.0)/5 (17.2)	6 (60.0)/24 (82.8)	Good vs. Fair–Poor	0.197	0.313	0.064–1.533
	*F. nucleatum*	3 (37.5)/6 (19.4)	5 (62.5)/25 (80.6)	Good vs. Fair–Poor	0.355	0.400	0.074–2.159
	*C. albicans*	3 (42.9)/6 (18.8)	4 (57.1)/26 (81.3)	Good vs. Fair–Poor	0.319	0.308	0.054–1.754

OR: odds ratio; CI: confidence interval; chi-squared test significant values (*P* < 0.05) are shown in bold

## Discussion

 The present findings revealed that *Aggregatibacter actinomycetemcomitans* was most abundant in subjects with Stage IV periodontitis. This aligns with earlier studies demonstrating that this bacterium produces quorum-sensing autoinducer-2 molecules in vitro, which interfere with *Candida albicans* hyphal formation and biofilm development.^16,17^ In addition, significant associations were observed between periodontitis severity and salivary IgA and IgG directed against *Fusobacterium nucleatum* and *C. albicans*. *F. nucleatum*, a representative of the orange complex, is an opportunistic pathogen well known for its adhesive capacity. By binding to various bacterial species, it acts as a bridge between commensals and pathogenic organisms within dental biofilms.^18^

 The role of orange complex bacteria is crucial for sustaining the red complex, as the latter depends on their presence for survival in the oral cavity.^19^ Adhesins such as FadA have intensified interest in the pathogenic role of *F. nucleatum*.^20^ Its involvement has been linked not only to periodontitis but also to multiple systemic disorders, including cardiovascular disease, adverse pregnancy outcomes, gastrointestinal infections, rheumatoid arthritis, diabetes mellitus, and gastric cancer, where it has even been suggested as a potential diagnostic marker.^21^ FadA facilitates binding to endothelial cells and promotes both pericellular and intracellular invasion, while also increasing endothelial permeability—mechanisms that may explain its systemic dissemination.^22^

 Although *F. nucleatum* is often susceptible to cytokine activity and phagocytosis, under certain conditions, it can stimulate inflammation through elevated production of proinflammatory cytokines and metalloproteinases. Molecules such as IL-8, MMP-9, and MMP-13 are particularly important in regulating migration and survival of infected epithelial cells.^23^ The bacterium can further modulate immunity by inducing apoptosis in circulating polymorphonuclear and mononuclear cells and impairing B- and T-lymphocyte function.^24^ Neutrophil function is also altered, with enhanced phagocytosis reported.^25^ Moreover, its serine proteases are able to degrade the IgA α-chain, allowing immune evasion.^26^

 Interestingly, the IgA and IgG responses to *F. nucleatum* in Stage IV cases were not markedly different from those in healthy controls. This parallels the work of Papapanou et al.,^27^ who observed that serum IgG titers against periodontal bacteria remained stable over extended periods and correlated with disease progression, though titers may temporarily decline following therapy. Host susceptibility, however, appears to be a critical factor in determining whether microbial imbalance progresses to destructive inflammation. Individuals able to tolerate the shift from symbiosis to dysbiosis may harbor dysbiotic microbiota without developing irreversible periodontal breakdown.^28^

 In the oral cavity, *C. albicans* colonizes mucosal surfaces, saliva, and periodontal pockets, suggesting a role in disease development.^29^ Its adaptability, including morphological switching between yeast and hyphal forms, contributes to survival and pathogenicity.^30^ Evidence from Canabarro et al.^31^ further supports its role in driving disease severity.^31^ In the present study, IgA and IgG reactivity to *C. albicans* increased with severity. Notably, *F. nucleatum* demonstrates strong adherence to *C. albicans*, mediated by carbohydrate–protein interactions between the two species.^32,33^

 Innate defenses usually prevent fungal overgrowth, including salivary flow, antimicrobial peptides like histatins, and competition with bacteria for nutrients. Activation of complement through alternative pathways, opsonization, and subsequent recognition by immune cells constitute another protective mechanism. Recognition of *C. albicans* pathogen-associated molecular patterns (PAMPs) by antigen-presenting cells via pattern recognition receptors, particularly TLR2 and TLR4, initiates both innate and adaptive immune cascades.^34^

 For *Porphyromonas gingivalis*, salivary IgA and IgG responses showed no association with severity. This observation supports the keystone pathogen hypothesis, which proposes that *P. gingivalis* manipulates the immune system to promote dysbiosis.^7^ Animal experiments confirm that low colonization levels of *P. gingivalis* can trigger inflammation and bone resorption only when accompanied by other microbes, as it fails to cause disease alone in germ-free rats.^35^ Virulence requires interaction with host immune receptors; strains lacking specific receptors cannot induce dysbiosis in mouse models.^7,36^ Gingipains, the Arg-specific cysteine proteases of *P. gingivalis*, activate complement C5a receptors and interact with TLR2, producing inflammatory responses while simultaneously impairing leukocyte bactericidal activity.^36,37^ Lipopolysaccharide variants further antagonize TLR4 pathways, reducing antimicrobial activity,^38^ while suppression of IL-8 synthesis prevents neutrophil recruitment to infection sites.^39^

 Serum studies indicate that IgA and IgG specific for *A. actinomycetemcomitans* are elevated in the early phases of systemic diseases, including cardiovascular disease, diabetes, and rheumatoid arthritis.^40-43^ In the present investigation, salivary IgG against *A. actinomycetemcomitans* was not clearly linked to severity, though stronger responses were noted in Stage IV patients. This concurs with Isola et al.,^43^ who reported higher serum IgG titers in periodontitis compared with healthy individuals.^43^ Gadekar et al.^44^ similarly observed higher serum and salivary IgA/IgG titers in chronic periodontitis patients.^44^ Importantly, correlations between *A. actinomycetemcomitans* levels and IgA/IgG responses were most pronounced in Stage III disease. One explanation may be that Stage III involves more affected teeth, whereas Stage IV is characterized by tooth loss, despite both stages sharing similar levels of clinical attachment loss.^45^

 Both plaque index and oral hygiene index increased significantly with disease severity. Dental plaque represents a multispecies biofilm adhering to tooth surfaces via host- and bacteria-derived pellicle receptors.^46,47^ Poor oral hygiene is an established risk factor, and a meta-analysis by Lertpimonchai et al.^48^ showed that inadequate oral hygiene increases the risk of periodontitis by two to five folds compared with good oral care.^48^ Papillary bleeding index, a marker of gingival inflammation, was likewise strongly associated with severity, supporting its clinical use as an indicator of host inflammatory response.^49^

 Among the pathogens examined, immune responses to *C. albicans* were most consistently correlated with clinical indices. Significant associations were demonstrated between plaque index and IgG against *C. albicans*, oral hygiene index and IgA against *C. albicans*, and papillary bleeding index and IgA against *C. albicans*. This is consistent with previous evidence that *C. albicans* biofilms create hypoxic microenvironments favoring anaerobic bacterial growth even under normally aerobic conditions.^50^

 Recent investigations have emphasized the potential use of salivary biomarkers, including pathogen-specific immunoglobulins, as adjunctive tools for periodontal disease assessment and monitoring.^51-53^ Integration of microbial detection with host immune mediators has been proposed to enhance disease stratification and improve understanding of host–microbe interactions in periodontitis.^52-54^ However, heterogeneity in study design, biomarker selection, and staging criteria has limited direct comparability across investigations. The present findings contribute to this evolving field by evaluating pathogen-specific IgA and IgG responses across severity stages defined according to the 2017 AAP/EFP classification. Unlike previous studies that evaluated isolated salivary biomarkers, the present study integrated pathogen-specific IgA and IgG responses with microbial detection across disease stages defined by the 2017 AAP/EFP classification, providing a stage-oriented immunological perspective of periodontitis severity.

 To our knowledge, this is the first investigation in Indonesia to evaluate the interplay between periodontal status (using the 2017 classification), salivary antibody responses against *A. actinomycetemcomitans*, *P. gingivalis*, *T. denticola*, *F. nucleatum*, and *C. albicans*, and the presence of these pathogens. Several limitations should be acknowledged. Although the sample size was determined a priori using an appropriate statistical formula, the relatively modest number of participants may still limit statistical power for subgroup analyses and broader generalizability. The cross-sectional design precludes assessment of longitudinal changes and prevents causal inference between microbial presence and salivary immune responses. In addition, grading of disease progression was not evaluated, and salivary findings were not directly compared with corresponding serum profiles. Potential confounding factors, including individual oral hygiene behavior and subclinical systemic conditions that may influence salivary composition, could not be fully controlled. Finally, inclusion of a broader spectrum of periopathogens and host-derived markers, such as cytokines and proteases, may further refine diagnostic and prognostic biomarker profiling in future investigations.

## Conclusion

 Salivary IgA and IgG responses to *Fusobacterium nucleatum* and *Candida albicans* demonstrate stage-related associations with periodontitis severity and selected clinical indices. These findings support the relevance of pathogen-specific humoral profiling as a complementary indicator of periodontal disease progression within the 2017 AAP/EFP classification framework. Further longitudinal studies are required to clarify their potential clinical applicability.

## Competing Interests

 The authors disclose no potential conflicts of interest affecting the authorship and/or publication of this article.

## Data Availability

 All methodological details of the manuscript have been provided.

## Ethical Approval

 This study was approved by the Ethics Committee of the Faculty of Dentistry, Universitas Indonesia (Approval No. 17/Ethical Approval/FKGUI/III/2019; Protocol No. 070210219).
